# Unusual Assortment of Segments in 2 Rare Human Rotavirus Genomes

**DOI:** 10.3201/eid1605.091826

**Published:** 2010-05

**Authors:** Simona De Grazia, Giovanni M. Giammanco, Christiaan A. Potgieter, Jelle Matthijnssens, Krisztián Bányai, Maria A. Platia, Claudia Colomba, Vito Martella

**Affiliations:** University of Palermo, Palermo, Italy (S. De Grazia, G.M. Giammanco, M.A. Platia, C. Colomba); Ondersterpoort Veterinary Institute, Ondersterpoort, South Africa (C.A. Potgieter); Rega Institute for Medical Research, Leuven, Belgium (J. Matthiinssens); Veterinary Medical Research Institute, Budapest, Hungary (K. Bányai); University of Bari, Valenzano, Italy (V. Martella)

**Keywords:** Viruses, zoonoses, rotavirus, genomes, G3P[9], full genome sequencing, genotyping, interspecies transmission, reassortment, dispatch

## Abstract

Using full-length genome sequence analysis, we investigated 2 rare G3P[9] human rotavirus strains isolated from children with diarrhea. The genomes were recognized as assortments of genes closely related to rotaviruses originating from cats, ruminants, and humans. Results suggest multiple transmissions of genes from animal to human strains of rotaviruses.

Group A rotaviruses possess a genome of 11 segments of double-stranded RNA (*1*). Rotaviruses are associated with acute gastroenteritis in humans and a wide variety of other mammalian and avian species (*1*). The evolution and diversity of rotaviruses is driven by genomic reassortment, accumulation of point mutations, intragenic recombination, and interspecies transmission (*2*,*3*). At least 23 G genotypes (structural viral protein [VP] 7 related) and 32 P genotypes (VP4 related) have been identified thus far in rotaviruses (*4*). Unlike other G and P types, G3 has been identified in rotavirus strains from humans and from almost all other susceptible mammalian species, including dogs, cats, monkeys, horses, rabbits, pigs, and ruminants, in association with various P types, thus exhibiting a broad host range (*1*). G3 human rotaviruses are usually associated with P[8] or P[6] and, rarely, with P[9] (5,*6*).

Historically, RNA–RNA hybridization has been used to study the genetic relationships among rotavirus strains and has shown 2 major pools among human rotaviruses, named Wa-like and DS-1–like (*7*). Recently, a new classification system based on whole-genome sequence analysis enabled researchers to better understand the complex interactions between human and animal rotaviruses (*8**,**9*). Application of this new classification system showed a close evolutionary relationship between human Wa-like and porcine rotavirus strains and between human DS-1–like and bovine rotavirus strains, suggesting that the 2 major human rotavirus G and P types might have an animal origin (*8*). A third human rotavirus family, designated AU-1–like, comprises a group of globally circulating but overall rare strains, mainly with the G3P[9] combination. Early RNA-RNA hybridization studies suggested a genetic relationship of particular human G3P[9] strains with feline rotaviruses (*10*). Later, feline–bovine reassortant G3P[9] rotaviruses were also identified in humans (*11*). However, because of the limits of resolution of the RNA–RNA hybridization method, determining the exact origin of individual genome segments in these strains was not possible.

## The Study

During uninterrupted surveillance for human rotaviruses in Palermo, Italy, which started in the mid-1980s, 3 strains (PAF96/94, PAH136/96, and PAI58/96) were detected that displayed AU-1–like features because they possessed long electropherotype, subgroup (SG) I (VP6 related) and G3P[9] genotypes. The viruses were identified from children <5 years of age who were hospitalized with acute gastroenteritis at the “G. Di Cristina” Children’s Hospital of Palermo in 1994 and 1996. Sequence analysis found all 3 strains to be genetically related to strains of either human or feline origin in the VP7, VP4, and VP6 genes. In contrast, the nonstructural protein (NSP) 4 gene of these viruses resembled that of G2P[4] human strains, suggesting a reassortment between AU-1–like and DS-1–like strains (*5*). To understand the evolution and origin of these viruses, we determined the full-length genome sequence of 2 such unusual G3P[9] viruses, strain PAH136/96 and PAI58/96, that appeared to be genetically distinct and for which enough material was available for additional analyses. The complete genome sequences were obtained as described elsewhere (*12*). Genome sequences were individually compared with cognate sequences of a variety of rotavirus strains by phylogenetic analysis by using MEGA4 software (www.megasoftware.net). In addition, the sequences were analyzed by using BLAST (www.ncbi.nlm.nih.gov/BLAST) with default search values. The GenBank nucleotide sequence accession numbers of PAH136/96 and PAI58/96, respectively, are GU296430 and GU296431 for VP7, GU296426 and GU296427 for VP4, GU296428 and GU296429 for VP6, GU296420 and GU296421 for VP1, GU296422 and GU296423 for VP2, GU296424 and GU296425 for VP3, GU296410 and GU296411 for NSP1, GU296412 and GU296413 for NSP2, GU296414 and GU296415 for NSP3, GU296416 and GU296417 for NSP4, GU296418 and GU296419 for NSP5.

The genomes of the PAH136/96 and PAI58/96 G3P[9] strains were 18,485 nt long. The 2 strains from Italy possessed the following genetic constellations: G3-P[9]-I2-R2-C2-M2-A3-N1-T6-E2-H3 for strain PAH136/96 and G3-P[9]-I2-R2-C2-M2-A3-N2-T6-E2-H3 for strain PAI58/96, differing only in the NSP2 gene (Tables 1, 2). Phylogenetic analysis showed that the VP2, VP3, VP4, VP7, NSP1, NSP3, NSP4, and NSP5 genomic segments of the 2 Italian G3P[9] viruses were closely related to each other, sharing high sequence similarity (Table 1; Technical Appendix). Although included in the same VP1 (R2) and VP6 (I2) genotypes, the 2 G3P[9] viruses in the VP1 and VP6 trees showed distinct patterns of segregation (Technical Appendix). Strains PAH136/96 and PAI58/96 shared only 83.2% nt and 86% nt identity in the VP1 and VP6 genes, respectively, i.e., values slightly above the proposed cutoff values for the VP1 (83%) and VP6 genotypes (85%) (Table 1).

**Table 1:** Nucleotide identity of 11 genome segments of 2 human rotavirus strains, Italy, 1994 and 1996*

Gene encoding	Cutoff value	Genotype of PAH136/96	Identity of PAH136/96 against indicated strains		Genotype of PAI58/96	Identity of PAI58/96 against indicated strains		Identity between PAH136/96 and PAI58/96
			Prototypes†	GenBank strains‡		Prototypes†	GenBank strains‡	
VP1	83	R2	83.7 (RF)	94.6 (Hun5)	R2	94.9 (RF)	95.7 (NCDV)	83.2
VP2	84	C2	90.9 (RF)	98 (Hun5)	C2	89.9 (RF)	93.9 (Chubut)	91.6
VP3	81	M2	89.2 (RF)	92.9 (PA169)	M2	90.2 (RF)	95.8 (PA169)	92.6
VP4	80	P[9]	95.6 (AU-1)	(AU-1)	P[9]	95.3 (AU-1)	(AU-1)	94.6
VP6	85	I2	86.9 (RF)	95.5 (Hun5)	I2	91.8 (RF)	92.4 (UKtc)	86
VP7	80	G3	90.1 (AU-1)	94.2 (Cat2)	G3	90.5 (AU-1)	95.9 (Cat2)	95.7
NSP1	79	A3	93.6 (AU-1)	94.8 (Chubut)	A3	92.9 (AU-1)	94.1 (Chubut)	92.1
NSP2	85	N1	91.4 (Wa)	99.1 (Cat2)	N2	90.1 (RF)	91.4 (NCDV)	79
NSP3	85	T6	94.7 (RF)	97 (MG6)	T6	95.9 (RF)	(RF)	97.5
NSP4	85	E2	87 (DS-1)	92.5 (PA169)	E2	87.5 (DS-1)	98 (PA169)	92.3
NSP5	91	H3	96.8 (RF)	97.3 (111/05)	H3	98 (RF)	98.8 (Cat2)	96.3

*Numeric values given as % nt. Percentage nucleotide cutoff values and genotype proposed by Matthijnssens et al. (*8*). VP, structural protein; NSP, nonstructural protein. †Prototype genotype strains used by Matthijnssens et al. (*8*). ‡ Strains that shared the highest nucleotide identity in the cognate genes with the Italian G3P[9] rotaviruses.

**Table 2:** Complete genomic constellations of the 2 G3P[9] Italian viruses sequenced together with several human P[14], ruminant, and feline rotaviruses and reference human strains Wa, DS-1, and AU-1*

Strain	VP7	VP4	VP6	VP1	VP2	VP3	NSP1	NSP2	NSP3	NSP4	NSP5
Hu/Ita/PA169	G6	P[14]	I2	R2	C2	M2	A3	N2	T6	E2	H3
Hu/Hung/Hun5	G6	P[14]	I2	R2	C2	M2	A11	N2	T6	E2	H3
Hu/B10925	G6	P[14]	I2	R2	C2	M2	A3	N2	T6	E2	H3
Hu/MG6	G6	P[14]	I2	R2	C2	M2	A11	N2	T6	E2	H3
Hu/111/05	G6	P[14]	I2	R2	C2	M2	A3	N2	T6	E2	H3
Ov/OVR762	G8	P[14]	I2	R2	C2	M2	A11	N2	T6	E2	H3
Gu/Arg/Chubut	G8	P[14]	I2	R5	C2	M2	A3	N2	T6	E2	H3
Bo/RF	G6	P[5]	I2	R2	C2	M2	A3	N2	T6	E2	H3
Bo/NCDV	G6	P[1]	I2	R2	C2	M2	?	N2	T6	E2	H3
**Hu/Ita/PAI58/96**	**G3**	**P[9]**	**I2**	**R2**	**C2**	**M2**	**A3**	**N2**	**T6**	**E2**	**H3**
**Hu/Ita/PAH136/96**	**G3**	**P[9]**	**I2**	**R2**	**C2**	**M2**	**A3**	**N1**	**T6**	**E2**	**H3**
Fe/Cat-2	G3	P[9]	I3	R3	C2	M3	A3	N1	T6	E3	H3
Fe/Cat-97	G3	P[3]	I3	R3	C2	M3	A9	N2	T3	E3	H6
Ca/CU-1	G3	P[3]	I3	R3	C2	M3	A9	N2	T3	E3	H6
Ca/Ro1845	G3	P[3]	I3	R3	C2	M3	A9	N2	T3	E3	H6
Ca/K9	G3	P[3]	I3	R3	C2	M3	A9	N2	T3	E3	H6
Ca/A79–10	G3	P[3]	I3	R3	C2	M3	A9	N2	T3	E3	H6
Hu/HCR3A	G3	P[3]	I3	R3	C2	M3	A9	N2	T3	E3	H6
Hu/AU-1	G3	P[9]	I3	R3	C3	M3	A3	N3	T3	E3	H3
Hu/DS-1	G2	P[4]	I2	R2	C2	M2	A2	N2	T2	E2	H2
Hu/Wa	G1	P[8]	I1	R1	C1	M1	A1	N1	T1	E1	H1

*Gray shading indicates genetic relationships with respect to the G3P[9] Italian viruses, according to the patterns of segregation displayed in the phylogenetic analyses in the Technical Appendix. **Boldface** indicates complete genomic constellations of the 2 G3P[9] Italian viruses sequenced in this study. VP, structural protein; NSP, nonstructural protein.

We found that after sequence and phylogenetic analysis, each of the 11 genomic segments of the Italian G3P[9] viruses had a striking genetic similarity with the corresponding segment of G6/G8P[14] human or ruminant rotaviruses or to human/feline AU-1–like rotaviruses (Table 1; Technical Appendix). In particular, the PAH136/96 strain possesses VP1, VP2, VP3, VP6, NSP3, NSP4, and NSP5 gene segments closely related to G6P[14] human rotaviruses, and the PAI58/96 strain possesses VP1, VP2, VP3, VP6, NSP2, NSP3, and NSP4 gene segments clustering with P[14] human rotaviruses, bovine rotaviruses, and other ruminant rotaviruses. The NSP1 genes of both strains segregated in a distinct branch within the A3 genotype containing feline, human/feline-like rotaviruses, and a G8P[14] rotavirus strain isolated from a guanaco (Technical Appendix). Matthijnssens et al. demonstrated that human G6P[14] rotaviruses were closely related to bovine and G6/G8P[14] ovine, antelope, and guanaco rotavirus strains because they shared a consensus genomic constellation (G6/G8)-P[14]-I2-(R2/R5)-C2-M2-(A3/A11)-N2-T6-(E2/E12)-H3 (*13*). The NSP2 gene of PAH136/96 and the NSP5 of PAI58/96 were strictly related to the cognate sequences of the feline Cat2 strain. The VP7 and VP4 genes of PAH136/96 and PAI58/96 human rotaviruses were also highly similar to the Cat2 strain and the feline/human-like AU-1 strain. The Cat2 strain displayed a puzzling genomic composition (G3/P[9]-I3-R3-C2-M3-A3-N1-T6-E3-H3), which we hypothesize resulted from multiple reassortment events involving canine, feline, human, and bovine rotaviruses (*14*). No genetic correlation was found in the VP7 gene between the G3P[9] strains of this study and the human G3 strains circulating in Palermo over a 20-year surveillance period (*15*).

## Conclusions

Full-genome sequencing of 2 unusual G3P[9] human rotavirus strains identified in Italy indicated that 1) viruses with a genetic makeup different from the Wa-, DS-1-, and AU-1-like gene pools may circulate in humans; 2) these viruses appear to have a relatively stable genetic constellation originating from reassortment events among feline/human AU-1–like rotaviruses, feline Cat2-like rotaviruses, and either ruminant rotaviruses or G6P[14] human rotaviruses; 3) these viruses, although retaining a stable genetic constellation, do not appear to have a clonal origin but are more likely to result from multiple introductions of particular genome segments from currently unknown animal rotavirus reservoirs.

Investigating the genetic features of human rotaviruses with unusual genetic/antigenic makeup is pivotal to gather information on the mechanisms by which some rotavirus strains may emerge in human populations. In addition, because of the possible animal origin of G3P[9] viruses, epidemiologic studies are warranted to identify the animal reservoir.

## Supplementary Material

Technical Appendix Phylogenetic trees based on the full-length nucleotide sequences of 11 rotavirus genes: viral proteins (VP) 1-5, 6, and 7, and nonstructural proteins (NSP) 1-5. The trees were generated by using the neighbor-joining method and Kimura 2-parameter model.
