# Supplementary material for: Unusual Assortment of Segments in 2 Rare Human Rotavirus Genomes
Source: Emerg Infect Dis. 2010 May;16(5):859–62. doi: 10.3201/eid1605.091826 (PMC2954010; doi:10.3201/eid1605.091826)
Supplement: Technical Appendix — Phylogenetic trees based on the full-length nucleotide sequences of 11 rotavirus genes: viral proteins (VP) 1-5, 6, and 7, and nonstructural proteins (NSP) 1-5. The trees were generated by using the neighbor-joining method and Kimura 2-parameter model. [file 09-1826_Techapp-s1.pdf]

Unusual Assortment of Segments in 2 Rare Human Rotavirus Genomes

Technical Appendix

Phylogenetic trees based on the full-length nucleotide sequences of 11 rotavirus genes: viral proteins (VP) 1–5, 6, and 7, and nonstructural proteins (NSP) 1–5. The trees were generated by using the neighbor-joining method and Kimura 2-parameter model. Bootstrap values (500 replicates) >80 are shown. The various genotypes are bo, bovine; hu, human; fe, feline; ca, canine; gu, guanacos; si, simian; ov, ovine; av, avian. The Italian G3P[9] viruses are in **boldface**. Scale bars indicate genetic distance between sequences of segments analyzed.

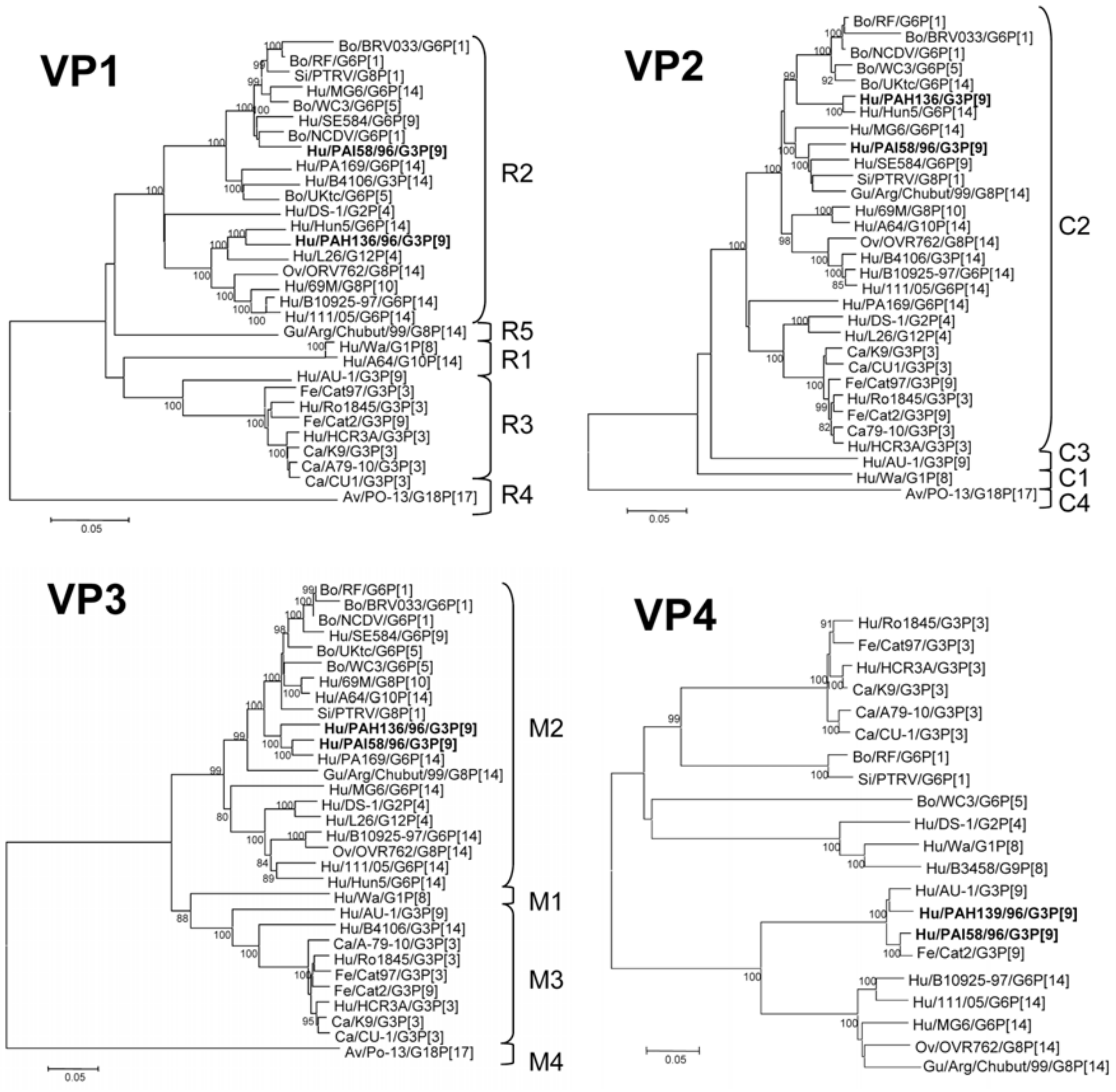

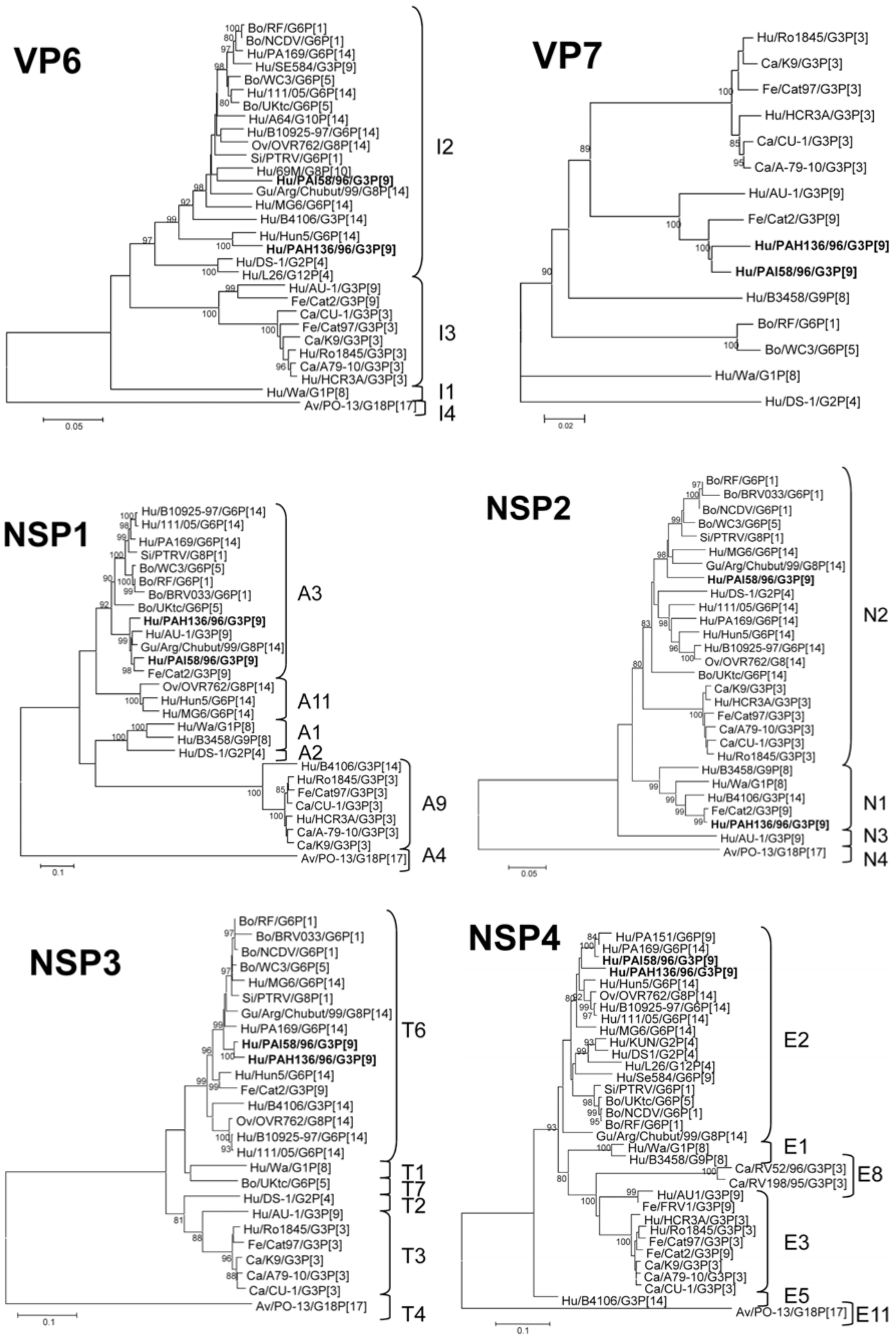
VP6
Bo/RF/G6P[1]
Bo/NCDV/G6P[1]
Hu/PA169/G6P[14]
Hu/SE584/G3P[9]
Bo/WC3/G6P[5]
Hu/111/05/G6P[14]
Bo/UKtc/G6P[5]
Hu/A64/G10P[14]
Hu/B10925-97/G6P[14]
Ov/OVR762/G8P[14]
Si/PTRV/G6P[1]
Hu/69M/G8P[10]
Hu/PAI58/96/G3P[9]
Gu/Arg/Chubut/99/G8P[14]
Hu/MG6/G6P[14]
Hu/B4106/G3P[14]
Hu/Hun5/G6P[14]
Hu/PAH136/96/G3P[9]
Hu/DS-1/G2P[4]
Hu/L26/G12P[4]
Hu/AU-1/G3P[9]
Fe/Cat2/G3P[9]
Ca/CU-1/G3P[3]
Fe/Cat97/G3P[3]
Ca/K9/G3P[3]
Hu/Ro1845/G3P[3]
Ca/A79-10/G3P[3]
Hu/HCR3A/G3P[3]
Hu/Wa/G1P[8]
Av/PO-13/G18P[17]
I2
I3
I1
I4
0.05
VP7
Hu/Ro1845/G3P[3]
Ca/K9/G3P[3]
Fe/Cat97/G3P[3]
Hu/HCR3A/G3P[3]
Ca/CU-1/G3P[3]
Ca/A-79-10/G3P[3]
Hu/AU-1/G3P[9]
Fe/Cat2/G3P[9]
Hu/PAH136/96/G3P[9]
Hu/PAI58/96/G3P[9]
Hu/B3458/G9P[8]
Bo/RF/G6P[1]
Bo/WC3/G6P[5]
Hu/Wa/G1P[8]
Hu/DS-1/G2P[4]
0.02
NSP1
Hu/B10925-97/G6P[14]
Hu/111/05/G6P[14]
Hu/PA169/G6P[14]
Si/PTRV/G8P[1]
Bo/WC3/G6P[5]
Bo/RF/G6P[1]
Bo/BRV033/G6P[1]
Bo/UKtc/G6P[5]
Hu/PAH136/96/G3P[9]
Hu/AU-1/G3P[9]
Gu/Arg/Chubut/99/G8P[14]
Hu/PAI58/96/G3P[9]
Fe/Cat2/G3P[9]
Ov/OVR762/G8P[14]
Hu/Hun5/G6P[14]
Hu/MG6/G6P[14]
Hu/Wa/G1P[8]
Hu/B3458/G9P[8]
Hu/DS-1/G2P[4]
Hu/B4106/G3P[14]
Hu/Ro1845/G3P[3]
Fe/Cat97/G3P[3]
Ca/CU-1/G3P[3]
Hu/HCR3A/G3P[3]
Ca/A-79-10/G3P[3]
Ca/K9/G3P[3]
Av/PO-13/G18P[17]
A3
A11
A1
A2
A9
A4
0.1
NSP2
Bo/RF/G6P[1]
Bo/BRV033/G6P[1]
Bo/NCDV/G6P[1]
Bo/WC3/G6P[5]
Si/PTRV/G8P[1]
Hu/MG6/G6P[14]
Gu/Arg/Chubut/99/G8P[14]
Hu/PAI58/96/G3P[9]
Hu/DS-1/G2P[4]
Hu/111/05/G6P[14]
Hu/PA169/G6P[14]
Hu/Hun5/G6P[14]
Hu/B10925-97/G6P[14]
Ov/OVR762/G8[14]
Bo/UKtc/G6P[14]
Ca/K9/G3P[3]
Hu/HCR3A/G3P[3]
Fe/Cat97/G3P[3]
Ca/A79-10/G3P[3]
Ca/CU-1/G3P[3]
Hu/Ro1845/G3P[3]
Hu/B3458/G9P[8]
Hu/Wa/G1P[8]
Hu/B4106/G3P[14]
Fe/Cat2/G3P[9]
Hu/PAH136/96/G3P[9]
Hu/AU-1/G3P[9]
Av/PO-13/G18P[17]
N2
N1
N3
N4
0.05
NSP3
Bo/RF/G6P[1]
Bo/BRV033/G6P[1]
Bo/NCDV/G6P[1]
Bo/WC3/G6P[5]
Hu/MG6/G6P[14]
Si/PTRV/G8P[1]
Gu/Arg/Chubut/99/G8P[14]
Hu/PA169/G6P[14]
Hu/PAI58/96/G3P[9]
Hu/PAH136/96/G3P[9]
Hu/Hun5/G6P[14]
Fe/Cat2/G3P[9]
Hu/B4106/G3P[14]
Ov/OVR762/G8P[14]
Hu/B10925-97/G6P[14]
Hu/111/05/G6P[14]
Hu/Wa/G1P[8]
Bo/UKtc/G6P[5]
Hu/DS-1/G2P[4]
Hu/AU-1/G3P[9]
Hu/Ro1845/G3P[3]
Fe/Cat97/G3P[3]
Ca/K9/G3P[3]
Ca/A79-10/G3P[3]
Ca/CU-1/G3P[3]
Av/PO-13/G18P[17]
T6
T1
T7
T2
T3
T4
0.1
NSP4
Hu/PA151/G6P[9]
Hu/PA169/G6P[14]
Hu/PAI58/96/G3P[9]
Hu/PAH136/96/G3P[9]
Hu/Hun5/G6P[14]
Ov/OVR762/G8P[14]
Hu/B10925-97/G6P[14]
Hu/111/05/G6P[14]
Hu/MG6/G6P[14]
Hu/KUN/G2P[4]
Hu/DS1/G2P[4]
Hu/L26/G12P[4]
Hu/Se584/G6P[9]
Si/PTRV/G6P[1]
Bo/UKtc/G6P[5]
Bo/NCDV/G6P[1]
Bo/RF/G6P[1]
Gu/Arg/Chubut/99/G8P[14]
Hu/Wa/G1P[8]
Hu/B3458/G9P[8]
Ca/RV52/96/G3P[3]
Ca/RV198/95/G3P[3]
Hu/AU1/G3P[9]
Fe/FRV1/G3P[9]
Hu/HCR3A/G3P[3]
Hu/Ro1845/G3P[3]
Fe/Cat97/G3P[3]
Fe/Cat2/G3P[9]
Ca/K9/G3P[3]
Ca/A79-10/G3P[3]
Ca/CU-1/G3P[3]
Hu/B4106/G3P[14]
Av/PO-13/G18P[17]
E2
E1
E8
E3
E5
E11
0.1

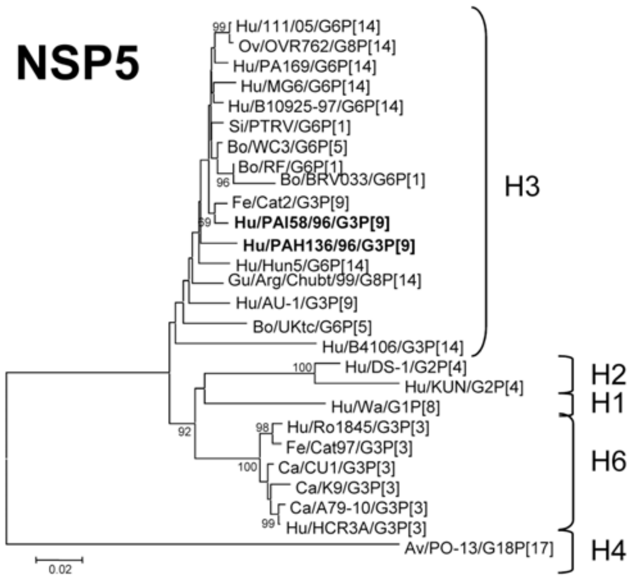
NSP5
Hu/111/05/G6P[14]
Ov/OVR762/G8P[14]
Hu/PA169/G6P[14]
Hu/MG6/G6P[14]
Hu/B10925-97/G6P[14]
Si/PTRV/G6P[1]
Bo/WC3/G6P[5]
Bo/RF/G6P[1]
Bo/BRV033/G6P[1]
Fe/Cat2/G3P[9]
Hu/PAI58/96/G3P[9]
Hu/PAH136/96/G3P[9]
Hu/Hun5/G6P[14]
Gu/Arg/Chubt/99/G8P[14]
Hu/AU-1/G3P[9]
Bo/UKtc/G6P[5]
Hu/B4106/G3P[14]
Hu/DS-1/G2P[4]
Hu/KUN/G2P[4]
Hu/Wa/G1P[8]
Hu/Ro1845/G3P[3]
Fe/Cat97/G3P[3]
Ca/CU1/G3P[3]
Ca/K9/G3P[3]
Ca/A79-10/G3P[3]
Hu/HCR3A/G3P[3]
Av/PO-13/G18P[17]
H3
H2
H1
H6
H4
0.02
